# RedDB, a computational database of electroactive molecules for aqueous redox flow batteries

**DOI:** 10.1038/s41597-022-01832-2

**Published:** 2022-11-28

**Authors:** Elif Sorkun, Qi Zhang, Abhishek Khetan, Murat Cihan Sorkun, Süleyman Er

**Affiliations:** DIFFER - Dutch Institute for Fundamental Energy Research, De Zaale 20, 5612 AJ Eindhoven, the Netherlands

**Keywords:** Energy, Computational chemistry, Cheminformatics, Scientific data

## Abstract

An increasing number of electroactive compounds have recently been explored for their use in high-performance redox flow batteries for grid-scale energy storage. Given the vast and highly diverse chemical space of the candidate compounds, it is alluring to access their physicochemical properties in a speedy way. High-throughput virtual screening approaches, which use powerful combinatorial techniques for systematic enumerations of large virtual chemical libraries and respective property evaluations, are indispensable tools for an agile exploration of the designated chemical space. Herein, RedDB: a computational database that contains 31,618 molecules from two prominent classes of organic electroactive compounds, quinones and aza-aromatics, has been presented. RedDB incorporates miscellaneous physicochemical property information of the compounds that can potentially be employed as battery performance descriptors. RedDB’s development steps, including: *(i)* chemical library generation, *(ii)* molecular property prediction based on quantum chemical calculations, *(iii)* aqueous solubility prediction using machine learning, and *(iv)* data processing and database creation, have been described.

## Abstract (summary)

**Table:** 

Measurement(s)	Clean Database • Physicochemical properties of redox active molecules • aqueous solubility
Technology Type(s)	Calculation • Physics • machine learning

## Background & Summary

The successful development of next-generation redox flow batteries with high cell voltage, energy density, and cycle life depends on the discovery of electroactive materials with optimum properties. Organic electroactive compounds have been attracting increasing attention due to their abundance, low cost, sustainable synthesis as well as recycling possibilities^1^. Notably, the compositional variance and structural diversity of electroactive compounds create plentiful opportunities for tuning their essential battery-relevant properties and thereby for their potential use as active battery materials. Given the nearly intractable configurational space of organic compounds, high-throughput virtual screening (HTVS) provides an effective way through, the creation of virtual libraries of diverse candidate electroactive compounds, computing performance-related chemical descriptors, prediction of molecular properties, and subsequent identification of the most promising candidates for further study^2^. The field of HTVS is burgeoning due to advances in automation of workflows and computing power, meanwhile the HTVS studies concerning the different classes of organic-based energy storage compounds are no exception^3–5^. HTVS generated FAIR data^6^, chiefly by employing accurate computational methods for the calculation of battery-relevant chemical descriptors, serves as a valuable reference for the advancement of aqueous redox flow battery (ARFB) technologies. Moreover, for an accelerated screening of the electroactive compound space for ARFBs, it is imperative to systematize the data in a way to make it accessible not only for humans and but also for machines.

In this work, we present a computational database, RedDB, that has been populated on a focused chemical space of candidate electroactive compounds as based on the two promising classes of ARFB molecules, namely, quinones^7–11^ and aza-aromatics^12–17^. RedDB is created by using the calculation data from physics-based simulation tools that employ molecular mechanics and quantum chemistry methods, in addition to the contemporary machine learning (ML) and cheminformatics generated data of the compounds. RedDB contains the predicted physicochemical properties of candidate molecules that are relevant to their function as electroactive components in ARFBs. Thus, it can be employed for material screening and/or empirical method development purposes.

**Fig. 1:**
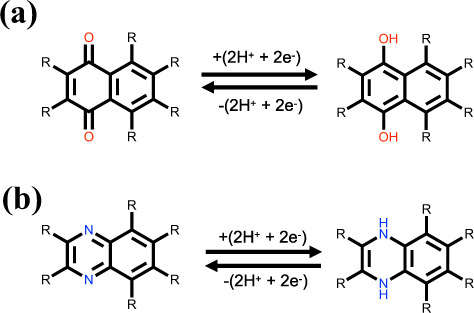
The reversible two-electron two-proton redox reactions that are shown for the two representative molecules of (**a**) quinone and (**b**) aza-aromatic. On the molecules, the positions that are employed for the systematic chemical functionalizations are shown with the R groups.

RedDB contains miscellaneous property data of the molecules, whilst the emphasis here is laid on the preeminent properties that relate to the redox potential. The thermodynamic basis to predict the redox potentials of electroactive compounds is the aqueous-phase redox reaction M + 2H^+^ + 2e^−^ ⇔ MH_2_, in which M is the electroactive molecular species. Accordingly for RedDB, M indicates either the quinone- or the aza-aromatic-derived reactant molecules, while MH_2_ indicates the corresponding hydrogenated product molecules that are generated through their respective chemical reactions shown in Fig. 1. The reaction energy, Δ*E*_rxn_, of redox couples has been calculated by using Eq. (1),

1 $$\Delta {E}_{{\rm{r}}{\rm{x}}{\rm{n}}}=E\left({{\rm{MH}}}_{2}\right)-\left[E\left({\rm{M}}\right)+E\left({{\rm{H}}}_{2}\right)\right],$$

where *E*(M), *E*(MH_2_), and *E*(H_2_) are the total energies of reactant and product molecules, and hydrogen molecules, respectively.

**Fig. 2:**
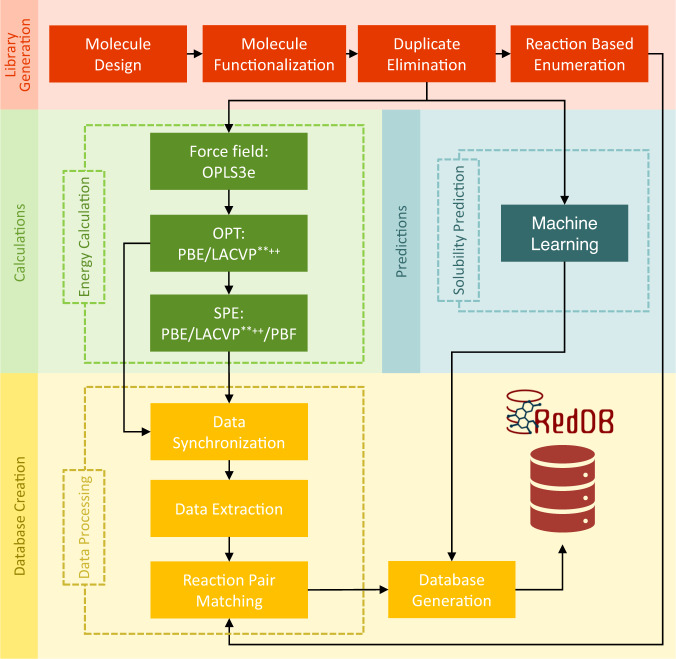
A schematic overview of the various tasks that have been undertaken for the development of RedDB. The three horizontal layers contain the main actions including, library generation (red shaded boxes), data generation, and database creation (yellow shaded boxes). The data generation includes both the electronic structure calculations (green shaded boxes) and the solubility predictions (blue shaded boxes). The boxes and arrows describe specific actions and flow of information, respectively.

RedDB’s building steps are outlined in Fig. 2. They include, virtual chemical library generation, physics-based calculations on molecules, ML predictions of solubility of compounds in water, and database creation. The systematic generation of the virtual library involves the creation of chemically functionalized derivatives of the reactant molecules and their redox reaction pair products. This step generates two-dimensional (2D) representations of all compounds in the virtual library, which are next used as inputs for both the first-principles calculations and the surrogate models. Accordingly, data generated from the two different types of methods is included in RedDB: (*i*) the electronic structure data that has been obtained from a sequence of classical and quantum chemical methods, and (*ii*) the aqueous solubility data that has been obtained by using a consensus ML model. In the last step of database development, the generated data is extracted, processed, and stored in a relational database by parsing the output files of the first-principles calculations and ML models.

RedDB has principally been built to support the design of new materials beyond conventional ARFB chemistries. It contains comprehensive data that has systematically been collected by using the state-of-the-art computational procedures^18,19^ and data-driven methods^20^. With an emphasis on the key properties of quinone- and aza-aromatic-based electroactive compounds, it contains several promising candidates with compelling predicted properties, which directly relate to the governing parameters for battery performance^21^, that are worthy of experimental investigation for practical use in ARFBs^22,23^. RedDB has been exported to five different data formats, as explained in the Usage Notes below, in order to serve the users who want to apply their own metrics in the most suitable format for them when they are working with RedDB data as a reference source. In addition to containing thus far uncharted depths of chemical space of small electroactive molecules and being a reference database for specialized studies on ARFBs, RedDB is also expected to be useful for other applications beyond ARFBs for which the intriguing chemistry of these molecules will matter. Identifying suitable redox-active molecules from the immense chemical space of small molecules requires rapid screening techniques with good precision in the predicted properties. However, due to the prohibitively costly computing requirements of robust quantum chemical simulations at large scale, it is not straightforward to scale-up HTVS efforts by orders of magnitude, such as from thousands to millions of molecules. RedDB, owing to its size, diversity, and quality of data, serves as a good resource for the development of empirical ML models that can be used for rapid property predictions or, more ambitiously, for the *de novo* design of energy compounds with desired features.

## Methods

RedDB was built in three stages, and by applying various methods within each stage, as described in below.

### Molecule library generation

**Fig. 3:**
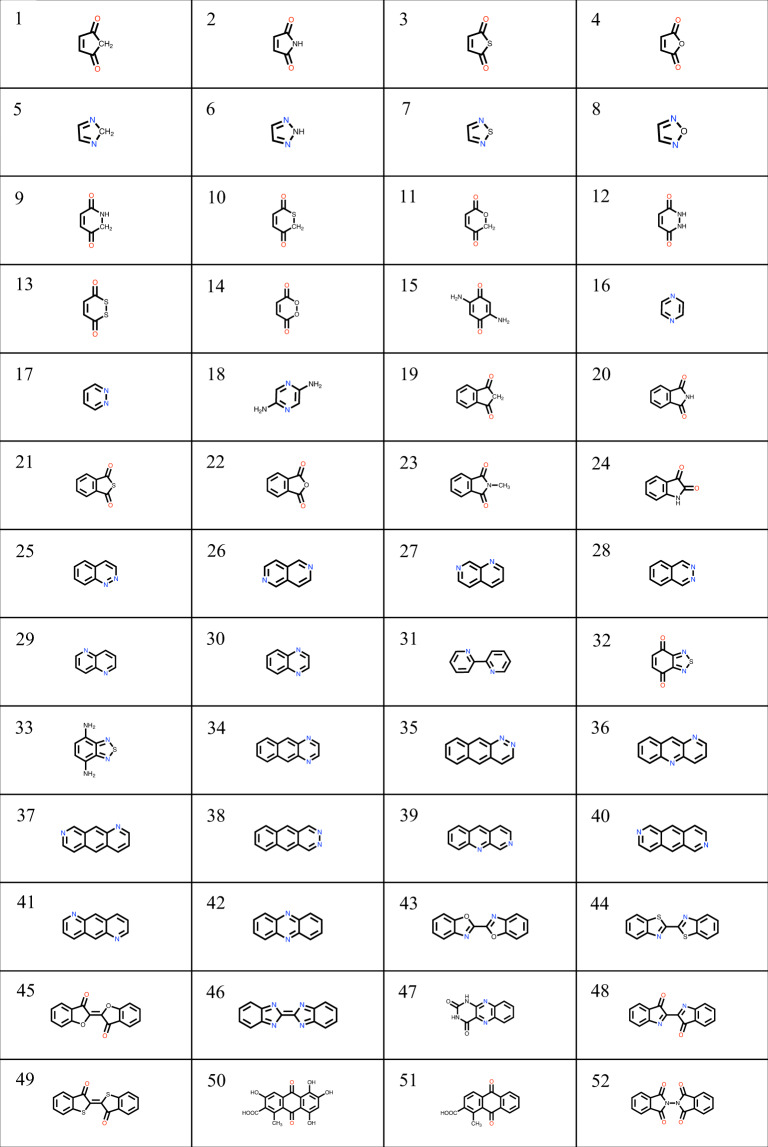
2D representations of the 52 core molecules that have been used for the chemical library generation. The numbering of the core molecules is in accordance with the data package IDs found in RedDB.

The steps of the library enumeration process are shown in Fig. 2. All the molecules in the virtual library were originally derived from a group of 24 quinone and 28 aza-aromatic reactant core structures that are deemed to be promising ARFB compounds in acidic or alkaline solutions (Fig. 3). The core molecule structures were designed manually by using the Maestro modeling interface of Schrödinger Materials Science Suite v2019-2 (SMSS)^24^. Next, the Custom R-group Enumeration tool of SMSS was employed to perform an exhaustive enumeration task in order to uncover all of the possible functionalized derivatives of the reactant core structures as well as their redox coupled product molecules. Five distinct R-groups (–SO_3_H, –﻿COOH, –﻿NH_2_, –﻿OH, and ﻿–F) were used for the chemical functionalization of compounds. These R-groups were decided upon the available chemical knowledge regarding their ability to tune the redox potential and aqueous solubility of the compounds^4^. It is known that incorporation of electron donating groups such as ﻿–OH and ﻿–NH_2_ decreases the electron affinity of the parent molecules and therefore usually results in lower redox potential values than their parent molecules. On the contrary, the use of electron-withdrawing groups such as ﻿–SO_3_H, –COOH, and –F leads to an opposite effect and results in functionalized molecules with higher redox potentials than their parent molecules. Additionally, functional groups such as ﻿–OH, ﻿–NH_2_, ﻿–COOH, and –﻿SO_3_H are known to improve the solubility of quinones^18^ and aza-aromatics^19^. In order to remove redundant entries of the generated molecules, the virtual library was screened by using the Filter Duplicates tool of SMSS. Also at this stage, the reactant-product molecule couples were paired by assuming a two-electron two-proton reaction mechanism^9^ shown in Fig. 1. We used the Reaction-based Enumeration tool of SMSS in order to match each reactant molecule to its corresponding product molecule. This way the enumeration process has been completed. It must be noted that both of the enumeration tools that were used in the current work accept the SMILES^25^ representations of molecules as their inputs. Therefore, they do not require explicit three-dimensional (3D) geometry information of the compounds. Similarly, the output format of these tools is also the SMILES representations. Therefore, when further evaluations on the molecules are aimed for, as the case of current study, they have to be translated to a 3D geometry data.

### Molecule structure and property data generation

#### Electronic structure calculations

First-principles electronic structure calculations yield essential information about the compounds that can directly be employed to estimate their macroscopic performance. Likewise, these calculations provide an effective way for the modelling of redox active compounds for ARFB applications^4,18,19^. Fig. 2 shows a simplified workflow of the physics-based calculations that were applied in the current work.

First, prior to quantum chemical calculations, the SMILES representations of all the candidate molecules that are found in the library were converted to energy minimized 3D molecular structures by using the LigPrep module as implemented in the Schrödinger Software Package^26^. Next, their corresponding minimum energy 3D conformers were predicted by using the MacroModel program and OPLS3e^27^ force field (FF) as implemented in SMSS. Thus, only the lowest energy 3D conformers were employed as inputs for density functional theory (DFT) calculations that were used for the gas phase optimization (OPT) of all molecules. Then, the DFT calculations were carried out using the Jaguar *ab initio* package^28^ as implemented in SMSS. All DFT calculations were performed using PBE exchange-correlation functional^29^ and LACVP^**++^ basis set with polarization and diffuse functions^30^. The LACVP basis set includes the effects of core electrons in a parametrized form known as the effective core potentials (ECPs). Using ECPs is advantageous, with regard to computing time, particularly when calculating compounds that contain many heavy elements. Moreover, LACVP and the widely employed 6-31 G basis sets are essentially indistinguishable for the elements from H to Ar. Since the molecules considered in this work contain only H, C, N, O, F, and S, the use of LACVP^**++^ is consistent with the use of 6-31 G^**++^. For DFT OPT calculations, medium grid densities have been used in Jaguar, and the energy and root mean square density matrix change convergence criteria were kept at their default values of 5.0 × 10^−5^ and 5.0 × 10^−6^ Hartree, respectively. As the convergence scheme, the default direct inversion in the iterative subspace was employed in combination with Jaguar’s mixed pseudospectral approximation at its default cutoffs. Lastly, the DFT optimized 3D geometries of the compounds were used as inputs for single point energy (SPE) calculations. For the SPE calculations, fine electronic grid densities, in combination with accurate self-consistent field cutoffs, were used. Additionally, the effects of aqueous medium were modelled by using the implicit Poisson-Boltzmann Solvation Model (PBF)^31^.

#### Solubility predictions

The water solubility data of the compounds was built by using the Aqueous Solubility Prediction Model (AqSolPred v1.0)^20^. AqSolPred is a supervised and consensus ML model that was empowered by training on a large, curated, and reference aqueous solubility database, AqSolDB^32^. The SMILES representations of the molecules were used as input for the AqSolPred and their ML-predicted solubility data has been incorporated to RedDB (Fig. 2).

### Database creation

Five different data sources were used as input for building the database: (1) Identifier files containing the SMILES representations of reactant and product molecules, (2) Supplementary files containing naming conventions for reactant molecules and SMILES notations of product molecules, (3) Output files as obtained from OPT calculations using the Jaguar package, (4) Output files as obtained from SPE calculations using the Jaguar package, and (5) Aqueous solubility data of compounds as obtained using the AqSolPred code.

The database creation process consists of data processing and database generation steps (Fig. 2). The former includes three steps, namely, data synchronization, data extraction, and chemical reaction pair matching. In the data synchronization step, the calculation output folder hierarchy and file naming conventions were created. By using them and the SMILES notations, the molecules from the virtual library were matched with the output files of the quantum chemical calculations. In the data extraction step, all output files were parsed by using an in-house developed code that employs regular expression sequences to extract relevant physicochemical data. In the chemical reaction pair matching step, the reactant molecules were matched with their respective products from the chemical library through the guidance of supplementary files that were generated by using the Reaction-based Enumeration tool^24^. In the database generation step, the database has been created on a MySQL server and implemented through a code first approach by using the Django object-relational mapper. Lastly, the parsed data, also including the ML-predicted solubility data of the compounds, has been added to RedDB.

## Data Records

The generated full data is stored in a MySQL database, and its reduced forms in CSV and XLSX formats, all of which are downloadable from the Harvard Dataverse Repository^33^. The data is stored in a relational database that consists of 15 data tables. These tables were created in accordance with the type of data they contain. Their names and brief descriptions as well as the original sources that have been used for their formulation are shown in Table 1.

**Table 1:** An overview of RedDB data tables.

Table Name	Table Description	Data Source
atomicProperties	Atomic properties from DFT SPE calculations (e.g. NMR shielding constants, Fukui indices for HOMO and LUMO, etc.)	Jaguar DFT SPE output file
chCalc	Moments from quantum mechanical wavefunction, electrostatic potential charges, and Mulliken charges (gas and solution phase)	Jaguar DFT SPE output file
cpolarCalc	Polarizability and hyperpolarizability results from coupled perturbed HF (cpolar) method	Jaguar DFT SPE output file
functionalGroup	Stoichiometric information on chemical functional groups	User-defined folder name
job	Meta-information of calculation outputs	Jaguar DFT OPT and SPE output files
jobSetting	Information on software version and calculation settings and parameters	Jaguar DFT OPT and SPE output files
molecule	Identifiers of molecules (SMILES and InChIKey)	SMILES output file
moleculeInfo	Stoichiometric information of the molecules	Jaguar DFT SPE output file
optimizationGeometry	Initial and final 3D geometries of molecules from DFT OPT	Jaguar DFT OPT output file
optimization	Convergence level and results from DFT OPT calculations	Jaguar DFT OPT output file
otherInfo	Additional information (e.g. nuclear repulsion energy, point group used for calculations, and molecular point group)	Jaguar DFT SPE output file
pbfCalc	Results from DFT SPE calculations with the PBF solvation model included	Jaguar DFT SPE output file
reaction	Redox reaction related information	Reaction-based Enumeration tool output file
solubility	ML-predicted solubility data of compounds	AqSolPred output file
scfCalc	Self-consistent field results from SPE calculations (gas and solution)	Jaguar DFT SPE output file

For each data table, the table name, a brief description of the contents, and the original data source from where the data has been extracted, are shown.

**Fig. 4:**
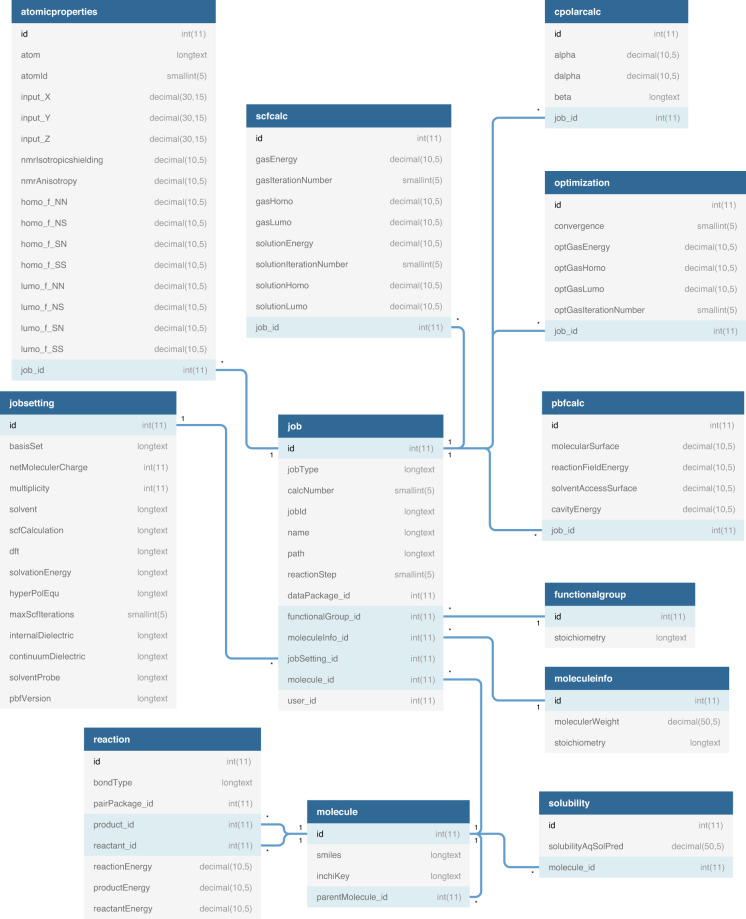
A *Crow’s foot* representation of RedDB’s most essential database tables.

RedDB contains data on 31,618 unique molecules that have been derived through the structural functionalization of 52 different core molecules shown in Fig. 3. For every compound, structural, thermodynamic, and electronic properties have been included. RedDB includes 23 atom-, 315 molecule-, four reaction-, and 19 simulation-related meta-information fields. Supplementary Information Table S1 shows RedDB’s most essential data tables that contain the most relevant information for application of molecules in ARFBs. For each data table shown in Supplementary Information Table S1, in addition to the names of data columns, their brief descriptions and the corresponding units, whenever applicable, have been included. Additionally, in Fig. 4, a simplified scheme of the database is shown that includes the most essential RedDB tables, their data fields and the interconnections. Finally, the contents of all the remaining RedDB data tables have been provided in Supplementary Information Table S2.

**Fig. 5:**
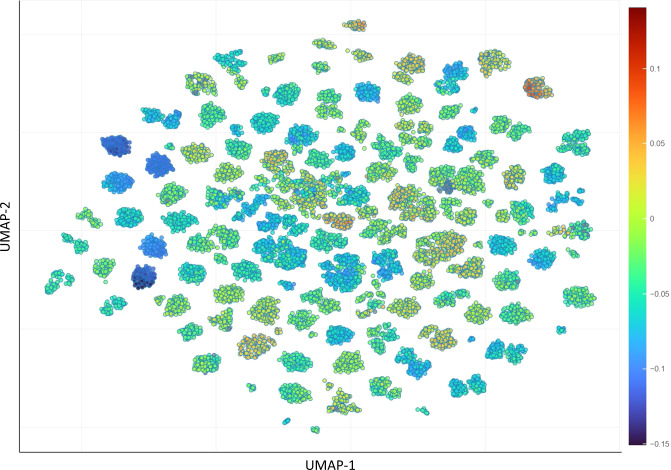
The chemical space of RedDB, as visualized by the ChemPlot using the UMAP dimensionality reduction technique in combination with the tailored similarity method. The color bar on the right shows the DFT-calculated Δ*E*_rxn_ values in Hartree as obtained by using  Eq. (1).

In RedDB, the total number of possible redox reactions, or similarly the reactant-product pairs, is 15,882. Among them are 3,509 quinone and 12,373 aza-aromatic molecule reactions. A mismatch between the total number of molecules and the total number of redox reactions occurs due to the molecules that take part in multiple redox reactions or the dismissed molecules because of failed DFT calculations. The reduced chemical space of RedDB’s chemical data, which has been converted into a visual representation via ChemPlot^34^ by applying the uniform manifold approximation and projection (UMAP) and tailored similarity methods, is shown in Fig. 5. Additionally, its interactive version is reachable at https://www.amdlab.nl/reddb.

## Technical Validation

The data in RedDB is generated from either first-principles or regression models, both of which are entirely parameterized. The data generated from such models is not stochastic and it is possible to reproduce it to numerical precision by using the parameters discussed above. In addition, reliability of the modelling data can also be interpreted in terms of its accuracy with respect to measurements from experiments. Thus, the sources of uncertainties are tied to the applied modelling parameters and the quality of underlying data. In the current work, to ensure the veracity of data that’s included in RedDB, several measures have been taken into account during the library generation process, DFT calculations, ML predictions, and database creation.

### Validation of library enumeration and convergence in DFT calculations

The molecule library generation included steps for filtering the duplicate molecules and removing the redundancies. To ensure that the molecular geometries employed for DFT OPT calculations are the lowest energy conformers, a sampling of the 3D conformational space of the molecules was performed. High accuracy for the two types of DFT calculations, OPT and SPE, was ensured by choosing tight convergence criteria for the various sub-routines in the Jaguar software package, as was described above. Further details on the systematic effect of these parameters can be found in the Jaguar documentation. Nevertheless, critical failures in convergence can result in spurious data that is unfit for further usage. To address this issue, Jaguar performs a simple analysis of the convergence during OPT, and yields a convenient verdict on the dynamics of the convergence process (i.e. whether the convergence was monotonic or erratic), and the quality of the converged structure (i.e. whether the final geometry corresponds to the lowest energy or not) on a scale of 0 to 4, where 0 denotes the best convergence. RedDB contains the convergence criteria for each molecule as obtained from OPT calculations employing the Jaguar package. This way, RedDB users are recommended to exercise caution when using data from molecules with convergence criteria value of 4, which simply indicates that the OPT resulted in a non-optimal structure of the molecule. In addition to this, DFT calculations on several molecules did not result in full convergence of the SCF routines, and thus, they did not produce any sensible results. Therefore, these molecules were also excluded from RedDB.

### Validation of solubility predictions

The AqSolPred model, which was used for solubility predictions in the current work, had previously been validated on a benchmark solubility dataset^35^. The model has a Mean Absolute Error of 0.348 LogS, which is lower than the conventional cheminformatics and ML methods that are ordinarily used for the prediction of aqueous solubility of chemical species^20^.

### Validation of data processing

The consistency of the data included in RedDB was further validated by comparing the values from randomly selected calculation output files to the data found in RedDB. For each of the 52 core molecule-derived groups of molecules, four randomly selected molecules’ DFT calculation output files have been used for comparisons. No consistency errors were detected on the cross-checked data.

## Usage Notes

Table 1 shows the names, descriptions, and data sources for each of the database tables. Additionally, the content descriptions and units of RedDB fields that are relevant to ARFBs are shown in Supplementary Information Table S1. The descriptions for the remaining tables are provided in Supplementary Information Table S2.

The ***‘job’*** table contains the parsed meta data of DFT OPT and DFT SPE calculation outputs. Thus, the results from both the OPT and SPE calculations are reachable simply by using ‘Optimization’ or ‘SinglePoint’ tags in the *‘jobType’* field in the ***‘job’*** table.

The ***‘job’*** and ***‘functionalGroup’*** tables are linked to each other with *‘functionalGroup id’*. Each identifier in the *‘functionalGroup id’* field represents a chemical functional group from the ***‘functionalGroup’*** table. A blank stoichiometry field in the ***‘functionalGroup’*** table indicates that no chemical functional group has been incorporated to the molecule, in other words, the molecule is a core molecule.

RedDB contains atomic, molecular, and reaction data of the candidate compounds for energy storage chiefly in ARFBs. To facilitate accessibility and reuse in future studies, RedDB has been exported to five different data formats that have been described in below.

### RedDB.sql

The file format is SQL. The relationships of database tables are shown in Fig. 4. The database tables are linked together by IDs. The content information of the tables has been provided in Supplementary Information Table S1 and Supplementary Information Table S2.

### RedDB.xlsx

The file format is XLSX. This file is a copy of the reddb.sql file. Each table of the database has been exported to a different sheet inside the XLSX file.

### RedDB_atomic.csv

The file format is CSV. This file contains all important atom properties of the molecules. Each row contains information on the atoms of a molecule. Using this file, the user can access all atom-relevant properties of the individual molecules, for instance by grouping the data according to the broadly accepted molecule identifiers of SMILES or InChIKey.

### RedDB_molecule.csv

The file format is CSV. This file contains all important molecule properties. Each row contains information on a single molecule.

### RedDB_reaction.csv

The file format is CSV. This file contains tabulated information about the likely redox reactions. Each row contains the reaction information and the DFT-calculated reaction energies. For the calculation of the reaction energies, the total energy of a H_2_ molecule was calculated by using the same methods that have been used for all other molecules. In addition to reaction energies^18^, other chemical descriptors, such as the lowest unoccupied molecular orbital (LUMO) of reactant and the highest occupied molecular orbital (HOMO) of product molecules, can independently be used to predict the experimental redox potentials^19^. For that reason, the numerical data of different chemical descriptors as well as useful compound features have also been included in this file.

## Supplementary information

Supplementary Information

## Data Availability

All classical and quantum chemical calculations have been performed by using the SMSS^24^, which is a proprietary software package. The solubility predictions have been made by using the AqSolPred^20^, which is a freely accessible tool. In addition, the in-house developed Python scripts that have been used to parse the calculation outputs and to convert them into relational database formats, are openly accessible at https://github.com/ergroup/RedDB.
