# Supplementary material for: RedDB, a computational database of electroactive molecules for aqueous redox flow batteries
Source: Sci Data. 2022 Nov 28;9:718. doi: 10.1038/s41597-022-01832-2 (PMC9705518; doi:10.1038/s41597-022-01832-2)
Supplement: Supplementary file 1 — Supplementary Information [file 41597_2022_1832_MOESM1_ESM.pdf]

Supplementary Information

RedDB, a computational database of electroactive molecules for aqueous redox flow batteries

Elif Sorkun[1], Qi Zhang[1], Abhishek Khetan[1], Murat Cihan Sorkun[1], and Süleyman Er[1]

Contents

1 Table S1 **2**

2 Table S2 **4**

[1]DIFFER – Dutch Institute for Fundamental Energy Research, De Zaale 20, 5612 AJ Eindhoven, the Netherlands.

1 Table S1

Table Name	Column Name	Column Description	Unit
17*atomicproperties	id	Unique ID for atomicproperties table	
	atom	Chemical element symbol	
	atomId	The ID of atom in the job	
	input_X	Numerical coordinate of atom position with reference to X axis	Å
	input_Y	Numerical coordinate of atom position with reference to Y axis	Å
	input_Z	Numerical coordinate of atom position with reference to Z axis	Å
	nmrIsotropicshielding	Isotropic NMR chemical shielding of the atom	ppm
	nmrAnisotropy	Anisotropic NMR chemical shielding of the atom	ppm
	homo_f_NN	f-NN-HOMO Fukui indice of the atom	Hartree
	homo_f_NS	f-NS-HOMO Fukui indice of the atom	Hartree
	homo_f_SN	f-SN-HOMO Fukui indice of the atom	Hartree
	homo_f_SS	f-SS-HOMO Fukui indice of the atom	Hartree
	lumo_f_NN	f-NN-LUMO Fukui indice of the atom	Hartree
	lumo_f_NS	f-NS-LUMO Fukui indice of the atom	Hartree
	lumo_f_SN	f-SN-LUMO Fukui indice of the atom	Hartree
	lumo_f_SS	f-SS-LUMO Fukui indice of the atom	Hartree
	job_id	Job ID of the calculation that the data was taken from	
5*cpolarcalc	id	Unique ID for cpolarcalc table	
	alpha	Polarizability quantity of α from cpolar calculation	au
	dalpha	Polarizability quantity of $\Delta\alpha$ from cpolar calculation	au
	beta	Average hyperpolarizability β from cpolar calculation	au
	job_id	Job ID of the calculation that the data was taken from	
2*functionalgroup	id	Unique ID for functionalgroup table	
	stoichiometry	Stoichiometry of functional groups	
13*job	id	Unique ID for job table	
	jobType	Type of calculation (SPE or OPT)	
	calcNumber	Calculation Number	
	jobId	Unique ID of the job as given by Jaguar	
	name	Name of the job file	
	path	Directory path of the job file	
	reactionStep	Reaction Step Number	
	dataPackage_id	ID of the data package with respective values shown in Fig. **??**	
	functionalGroup_id	Chemical functional group ID	
	jobSetting_id	Job settings ID	
	molecule_id	Molecule ID	
	moleculeInfo_id	Molecule info ID	
	user_id	User ID	
14*jobsetting	id	Unique ID for jobsetting table	
	basisSet	Basis set	
	netMoleculerCharge	Net molecular charge	e
	multiplicity	Multiplicity	
	solvent	Solvent information	
	scfCalculation	SCF calculation information	
	dft	DFT calculation information	
	solvationEnergy	Solvation method calculation information	
	hyperPolEqu	(Hyper) polarizabilities equation name	
	maxScfIterations	Maximum number of SCF iterations	
	internalDielectric	Internal dielectric constant	
	continuumDielectric	Continuum dielectric constant	
	solventProbe	Solvent probe molecule radius	Å^2
	pbfVersion	PBF model version information	
4*molecule	id	Unique ID for molecule table	
	smiles	SMILES representation of the molecule	
	inchiKey	InChIKey representation of the molecule	

	parentMolecule_id	Molecule ID of the parent (core) molecule	
3*moleculeinfo	id	Unique ID for moleculeinfo table	
	moleculerWeight	Total weight of the molecule	amu
	stoichiometry	Composition stoichiometry of the molecule	
7*optimization	id	Unique ID for reaction table	
	convergence	The quality of the converged structure (i.e. 0,1,2,3,4)	
	optGasEnergy	Total energy of the molecule in gas phase from OPT	Hartree
	optGasHomo	HOMO energy of the molecule in gas phase from OPT	Hartree
	optGasLumo	LUMO energy of the molecule in gas phase from OPT	Hartree
	optGasIterationNumber	Number of SCF iterations for gas phase calculation from OPT	
	job_id	Job ID of the calculation that the data was taken from	
6*pbfcalc	id	Unique ID for pbfcalc table	
	job_id	Job ID of the calculation that the data was taken from	
	cavityEnergy	Cavity energy of the molecule	kT
	molecularSurface	Molecule surface area	$Å^2$
	reactionFieldEnergy	Reaction field energy	kT
	solventAccessSurface	Solvent accessible surface	$Å^2$
8*reaction	id	Unique ID for reaction table	
	bondType	Type of redox reaction	
	pairPackage_id	ID of the data package containing reaction information	
	product_id	Product molecule ID of the redox reaction	
	reactant_id	Reactant molecule ID of the redox reaction	
	reactionEnergy	Reaction energy of the redox reaction	Hartree
	productEnergy	Total energy of the product molecule	Hartree
	reactantEnergy	Total energy of the reactant molecule	Hartree
3*solubility	id	Unique ID for solubility table	
	molecule_id	Molecule ID	
	solubilityAqSolPred	Predicted solubility value	logS
10*scfcalc	id	Unique ID for scfcalc table	
	gasEnergy	Total energy of the molecule in gas phase from SPE	Hartree
	gasIterationNumber	Number of SCF iterations for gas phase calculation from SPE	
	gasHomo	HOMO energy of the molecule in gas phase from SPE	Hartree
	gasLumo	LUMO energy of the molecule in gas phase from SPE	Hartree
	solutionEnergy	Total energy of the molecule in solution phase from SPE	Hartree
	solutionIterationNumber	Number of SCF iterations for solution phase calculation from SPE	
	solutionHomo	HOMO energy of the molecule in solution phase from SPE	Hartree
	solutionLumo	LUMO energy of the molecule in solution phase from SPE	Hartree
	job_id	Job ID of the calculation that the data was taken from	

Table S1: RedDB's most essential data tables. The names and brief descriptions of the data columns, which are specific to each data table, have been provided. In addition, for every data column, the applicable units for the stored data have been shown.

2 Table S2

Table Name	Column Name	Column Description	Unit
otherinfo	id	Unique ID for otherinfo table	
	jobId_id	Job ID of the calculation that the data was taken from	
	molecularPointGroup	Molecular Point Group	
	nuclearRepulsionEnergy	Nuclear Repulsion Energy	Hartree
	pointGroupUsed	Point Group Used	
optimizationgeometry	id	Unique ID for optimizationgeometry table	
	jobId_id	Job ID of the calculation that the data was taken from	
	atom	Chemical element symbol	
	atomId	The ID of the atom in the job	
	input_X	Numerical coordinate of atom position with reference to X axis	Å
	input_Y	Numerical coordinate of atom position with reference to Y axis	Å
	input_Z	Numerical coordinate of atom position with reference to Z axis	Å
	final_X	Optimized numerical coordinate of atom position with reference to X axis	Å
	final_Y	Optimized numerical coordinate of atom position with reference to Y axis	Å
	final_Z	Optimized numerical coordinate of atom position with reference to Z axis	Å
chcalc	id	Unique ID for chcalc table	
	jobId_id	Job ID of the calculation that the data was taken from	
	mqmwGasPossibleMaximum	Gridpoints possible maximum in gas phase	
	mqmwGasGridpointsChargeFit	Gridpoints used for charge fit in gas phase	
	mqmwPossibleMaximum	Gridpoints possible maximum in solution phase	
	mqmwGridpointsChargeFit	Gridpoints used for charge fit in solution phase	
	mqmwGasDipoleMomentsTot	Total dipole moments from quantum mechanical wavefunction in gas phase	Debye
	mqmwGasDipoleMomentsX	X dipole moments from quantum mechanical wavefunction in gas phase	Debye
	mqmwGasDipoleMomentsY	Y dipole moments from quantum mechanical wavefunction in gas phase	Debye
	mqmwGasDipoleMomentsZ	Z dipole moments from quantum mechanical wavefunction in gas phase	Debye
	mqmwGasQuadrupoleMomentsXX	XX quadrupole moments from quantum mechanical wavefunction in gas phase	Debye-Å
	mqmwGasQuadrupoleMomentsXY	XY quadrupole moments from quantum mechanical wavefunction in gas phase	Debye-Å
	mqmwGasQuadrupoleMomentsXZ	XZ quadrupole moments from quantum mechanical wavefunction in gas phase	Debye-Å
	mqmwGasQuadrupoleMomentsYY	YY quadrupole moments from quantum mechanical wavefunction in gas phase	Debye-Å
	mqmwGasQuadrupoleMomentsYZ	YZ quadrupole moments from quantum mechanical wavefunction in gas phase	Debye-Å
	mqmwGasQuadrupoleMomentsZZ	ZZ quadrupole moments from quantum mechanical wavefunction in gas phase	Debye-Å

mqmwGasTracelessQuadrupoleXY	XY traceless quadrupole moments from quantum mechanical wavefunction in gas phase	Debye-Å
mqmwGasTracelessQuadrupoleXZ	XZ traceless quadrupole moments from quantum mechanical wavefunction in gas phase	Debye-Å
mqmwGasTracelessQuadrupoleYZ	YZ traceless quadrupole moments from quantum mechanical wavefunction in gas phase	Debye-Å
mqmwGasTracelessQuadrupoleXXYY	XX-YY traceless quadrupole moments from quantum mechanical wavefunction in gas phase	Debye-Å
mqmwGasTracelessQuadrupol2ZZXXYY	2ZZ-XX-YY traceless quadrupole moments from quantum mechanical wavefunction in gas phase	Debye-Å
mqmwGasOctapoleMomentsXXX	XXX octapole moments from quantum mechanical wavefunction in gas phase	Debye-$Å^2$
mqmwGasOctapoleMomentsXXY	XXY octapole moments from quantum mechanical wavefunction in gas phase	Debye-$Å^2$
mqmwGasOctapoleMomentsXXZ	XXZ octapole moments from quantum mechanical wavefunction in gas phase	Debye-$Å^2$
mqmwGasOctapoleMomentsXYY	XYY octapole moments from quantum mechanical wavefunction in gas phase	Debye-$Å^2$
mqmwGasOctapoleMomentsXYZ	XYZ octapole moments from quantum mechanical wavefunction in gas phase	Debye-$Å^2$
mqmwGasOctapoleMomentsXZZ	XZZ octapole moments from quantum mechanical wavefunction in gas phase	Debye-$Å^2$
mqmwGasOctapoleMomentsYYY	YYY octapole moments from quantum mechanical wavefunction in gas phase	Debye-$Å^2$
mqmwGasOctapoleMomentsYYZ	YYZ octapole moments from quantum mechanical wavefunction in gas phase	Debye-$Å^2$
mqmwGasOctapoleMomentsYZZ	YZZ octapole moments from quantum mechanical wavefunction in gas phase	Debye-$Å^2$
mqmwGasOctapoleMomentsZZZ	ZZZ octapole moments from quantum mechanical wavefunction in gas phase	Debye-$Å^2$
mqmwGasTracelessOctapoleXXX	XXX traceless octapole moments from quantum mechanical wavefunction in gas phase	Debye-$Å^2$
mqmwGasTracelessOctapoleXYZ	XYZ traceless octapole moments from quantum mechanical wavefunction in gas phase	Debye-$Å^2$
mqmwGasTracelessOctapoleYYY	YYY traceless octapole moments from quantum mechanical wavefunction in gas phase	Debye-$Å^2$
mqmwGasTracelessOctapoleZZZ	ZZZ traceless octapole moments from quantum mechanical wavefunction in gas phase	Debye-$Å^2$
mqmwGasTracelessOctapoleXXYYZZ	XXY-YZZ traceless octapole moments from quantum mechanical wavefunction in gas phase	Debye-$Å^2$
mqmwGasTracelessOctapoleXXZYYZ	XXZ-YYZ traceless octapole moments from quantum mechanical wavefunction in gas phase	Debye-$Å^2$
mqmwGasTracelessOctapoleXYYXZZ	XYY-XZZ traceless octapole moments from quantum mechanical wavefunction in gas phase	Debye-$Å^2$
mqmwGasHexadecapoleMomentsXXXX	XXXX hexadecapole moments from quantum mechanical wavefunction in gas phase	Debye-$Å^3$
mqmwGasHexadecapoleMomentsXXXY	XXXY hexadecapole moments from quantum mechanical wavefunction in gas phase	Debye-$Å^3$
mqmwGasHexadecapoleMomentsXXXZ	XXXZ hexadecapole moments from quantum mechanical wavefunction in gas phase	Debye-$Å^3$

mqmwGasHexadecapoleMomentsXXYY	XXYY hexadecapole moments from quantum mechanical wavefunction in gas phase	Debye-Å^3
mqmwGasHexadecapoleMomentsXXYZ	XXYZ hexadecapole moments from quantum mechanical wavefunction in gas phase	Debye-Å^3
mqmwGasHexadecapoleMomentsXXZZ	XXZZ hexadecapole moments from quantum mechanical wavefunction in gas phase	Debye-Å^3
mqmwGasHexadecapoleMomentsYYXZ	YYXZ hexadecapole moments from quantum mechanical wavefunction in gas phase	Debye-Å^3
mqmwGasHexadecapoleMomentsYYYX	YYYX hexadecapole moments from quantum mechanical wavefunction in gas phase	Debye-Å^3
mqmwGasHexadecapoleMomentsYYYY	YYYY hexadecapole moments from quantum mechanical wavefunction in gas phase	Debye-Å^3
mqmwGasHexadecapoleMomentsYYYZ	YYYZ hexadecapole moments from quantum mechanical wavefunction in gas phase	Debye-Å^3
mqmwGasHexadecapoleMomentsYYZZ	YYZZ hexadecapole moments from quantum mechanical wavefunction in gas phase	Debye-Å^3
mqmwGasHexadecapoleMomentsZZXY	ZZXY hexadecapole moments from quantum mechanical wavefunction in gas phase	Debye-Å^3
mqmwGasHexadecapoleMomentsZZZX	ZZZX hexadecapole moments from quantum mechanical wavefunction in gas phase	Debye-Å^3
mqmwGasHexadecapoleMomentsZZZY	ZZZY hexadecapole moments from quantum mechanical wavefunction in gas phase	Debye-Å^3
mqmwGasHexadecapoleMomentsZZZZ	ZZZZ hexadecapole moments from quantum mechanical wavefunction in gas phase	Debye-Å^3
mqmwDipoleMomentsTot	Total dipole moments from quantum mechanical wavefunction in solution phase	Debye
mqmwDipoleMomentsX	X dipole moments from quantum mechanical wavefunction in solution phase	Debye
mqmwDipoleMomentsY	Y dipole moments from quantum mechanical wavefunction in solution phase	Debye
mqmwDipoleMomentsZ	Z dipole moments from quantum mechanical wavefunction in solution phase	Debye
mqmwQuadrupoleMomentsXX	XX quadrupole moments from quantum mechanical wavefunction in solution phase	Debye-Å
mqmwQuadrupoleMomentsXY	XY quadrupole moments from quantum mechanical wavefunction in solution phase	Debye-Å
mqmwQuadrupoleMomentsXZ	XZ quadrupole moments from quantum mechanical wavefunction in solution phase	Debye-Å
mqmwQuadrupoleMomentsYY	YY quadrupole moments from quantum mechanical wavefunction in solution phase	Debye-Å
mqmwQuadrupoleMomentsYZ	YZ quadrupole moments from quantum mechanical wavefunction in solution phase	Debye-Å
mqmwQuadrupoleMomentsZZ	ZZ quadrupole moments from quantum mechanical wavefunction in solution phase	Debye-Å
mqmwTracelessQuadrupoleXY	XY traceless quadrupole moments from quantum mechanical wavefunction for solution	Debye-Å
mqmwTracelessQuadrupoleXZ	XZ traceless quadrupole moments from quantum mechanical wavefunction for solution	Debye-Å
mqmwTracelessQuadrupoleYZ	YZ traceless quadrupole moments from quantum mechanical wavefunction for solution	Debye-Å
mqmwTracelessQuadrupoleXXYY	XX-YY traceless quadrupole moments from quantum mechanical wavefunction for solution	Debye-Å
mqmwTracelessQuadrupol2ZZXXYY	2ZZ-XX-YY traceless quadrupole moments from quantum mechanical wavefunction for solution	Debye-Å

mqmwOctapoleMomentsXXX	XXX octapole moments from quantum mechanical wavefunction in solution phase	Debye-Å^2
mqmwOctapoleMomentsXXY	XXY octapole moments from quantum mechanical wavefunction in solution phase	Debye-Å^2
mqmwOctapoleMomentsXXZ	XXZ octapole moments from quantum mechanical wavefunction in solution phase	Debye-Å^2
mqmwOctapoleMomentsXYY	XYY octapole moments from quantum mechanical wavefunction in solution phase	Debye-Å^2
mqmwOctapoleMomentsXYZ	XYZ octapole moments from quantum mechanical wavefunction in solution phase	Debye-Å^2
mqmwOctapoleMomentsXZZ	XZZ octapole moments from quantum mechanical wavefunction in solution phase	Debye-Å^2
mqmwOctapoleMomentsYYY	YYY octapole moments from quantum mechanical wavefunction in solution phase	Debye-Å^2
mqmwOctapoleMomentsYYZ	YYZ octapole moments from quantum mechanical wavefunction in solution phase	Debye-Å^2
mqmwOctapoleMomentsYZZ	YZZ octapole moments from quantum mechanical wavefunction in solution phase	Debye-Å^2
mqmwOctapoleMomentsZZZ	ZZZ octapole moments from quantum mechanical wavefunction in solution phase	Debye-Å^2
mqmwTracelessOctapoleXXX	XXX traceless octapole moments from quantum mechanical wavefunction in solution phase	Debye-Å^2
mqmwTracelessOctapoleYYY	YYY traceless octapole moments from quantum mechanical wavefunction in solution phase	Debye-Å^2
mqmwTracelessOctapoleZZZ	ZZZ traceless octapole moments from quantum mechanical wavefunction in solution phase	Debye-Å^2
mqmwTracelessOctapoleXYZ	XYZ traceless octapole moments from quantum mechanical wavefunction in solution phase	Debye-Å^2
mqmwTracelessOctapoleXXYYZZ	XXY-YZZ traceless octapole moments from quantum mechanical wavefunction in solution phase	Debye-Å^2
mqmwTracelessOctapoleXXZYYZ	XXZ-YYZ traceless octapole moments from quantum mechanical wavefunction in solution phase	Debye-Å^2
mqmwTracelessOctapoleXYYXZZ	XYY-XZZ traceless octapole moments from quantum mechanical wavefunction in solution phase	Debye-Å^2
mqmwHexadecapoleMomentsXXXX	XXXX hexadecapole moments from quantum mechanical wavefunction in solution phase	Debye-Å^3
mqmwHexadecapoleMomentsXXXY	XXXY hexadecapole moments from quantum mechanical wavefunction in solution phase	Debye-Å^3
mqmwHexadecapoleMomentsXXXZ	XXXZ hexadecapole moments from quantum mechanical wavefunction in solution phase	Debye-Å^3
mqmwHexadecapoleMomentsXXYY	XXYY hexadecapole moments from quantum mechanical wavefunction in solution phase	Debye-Å^3
mqmwHexadecapoleMomentsXXYZ	XXYZ hexadecapole moments from quantum mechanical wavefunction in solution phase	Debye-Å^3
mqmwHexadecapoleMomentsXXZZ	XXZZ hexadecapole moments from quantum mechanical wavefunction in solution phase	Debye-Å^3

mqmwHexadecapoleMomentsYYXZ	YYXZ hexadecapole moments from quantum mechanical wavefunction in solution phase	Debye-Å^3
mqmwHexadecapoleMomentsYYYX	YYYX hexadecapole moments from quantum mechanical wavefunction in solution phase	Debye-Å^3
mqmwHexadecapoleMomentsYYYY	YYYY hexadecapole moments from quantum mechanical wavefunction in solution phase	Debye-Å^3
mqmwHexadecapoleMomentsYYYZ	YYYZ hexadecapole moments from quantum mechanical wavefunction in solution phase	Debye-Å^3
mqmwHexadecapoleMomentsYYZZ	YYZZ hexadecapole moments from quantum mechanical wavefunction in solution phase	Debye-Å^3
mqmwHexadecapoleMomentsZZXY	ZZXY hexadecapole moments from quantum mechanical wavefunction in solution phase	Debye-Å^3
mqmwHexadecapoleMomentsZZZX	ZZZX hexadecapole moments from quantum mechanical wavefunction in solution phase	Debye-Å^3
mqmwHexadecapoleMomentsZZZY	ZZZY hexadecapole moments from quantum mechanical wavefunction in solution phase	Debye-Å^3
mqmwHexadecapoleMomentsZZZZ	ZZZZ hexadecapole moments from quantum mechanical wavefunction in solution phase	Debye-Å^3
mepcGasDipoleMomentsTot	Total dipole moment from electrostatic potential charges in gas phase	Debye
mepcGasDipoleMomentsX	X dipole moment from electrostatic potential charges in gas phase	Debye
mepcGasDipoleMomentsY	Y dipole moment from electrostatic potential charges in gas phase	Debye
mepcGasDipoleMomentsZ	Z dipole moment from electrostatic potential charges in gas phase	Debye
mepcGasQuadrupoleMomentsXX	XX quadrupole moment from electrostatic potential charges in gas phase	Debye-Å
mepcGasQuadrupoleMomentsXY	XY quadrupole moment from electrostatic potential charges in gas phase	Debye-Å
mepcGasQuadrupoleMomentsXZ	XZ quadrupole moment from electrostatic potential charges in gas phase	Debye-Å
mepcGasQuadrupoleMomentsYY	YY quadrupole moment from electrostatic potential charges in gas phase	Debye-Å
mepcGasQuadrupoleMomentsYZ	YZ quadrupole moment from electrostatic potential charges in gas phase	Debye-Å
mepcGasQuadrupoleMomentsZZ	ZZ quadrupole moment from electrostatic potential charges in gas phase	Debye-Å
mepcGasTracelessQuadrupoleXY	XY traceless quadrupole moment from electrostatic potential charges in gas phase	Debye-Å
mepcGasTracelessQuadrupoleXZ	XZ traceless quadrupole moment from electrostatic potential charges in gas phase	Debye-Å
mepcGasTracelessQuadrupoleYZ	YZ traceless quadrupole moment from electrostatic potential charges in gas phase	Debye-Å
mepcGasTracelessQuadrupoleXXYY	XX-YY traceless quadrupole moment from electrostatic potential charges in gas phase	Debye-Å
mepcGasTracelessQuadrupol2ZZXXYY	2ZZ-XX-YY traceless quadrupole moment from electrostatic potential charges in gas phase	Debye-Å
mepcGasOctapoleMomentsXXX	XXX octapole moment from electrostatic potential charges in gas phase	Debye-Å^2
mepcGasOctapoleMomentsXXY	XXY octapole moment from electrostatic potential charges in gas phase	Debye-Å^2
mepcGasOctapoleMomentsXXZ	XXZ octapole moment from electrostatic potential charges in gas phase	Debye-Å^2

mepcGasOctapoleMomentsXYY	XYY octapole moment from electrostatic potential charges in gas phase	Debye-Å^2
mepcGasOctapoleMomentsXYZ	XYZ octapole moment from electrostatic potential charges in gas phase	Debye-Å^2
mepcGasOctapoleMomentsXZZ	XZZ octapole moment from electrostatic potential charges in gas phase	Debye-Å^2
mepcGasOctapoleMomentsYYY	YYY octapole moment from electrostatic potential charges in gas phase	Debye-Å^2
mepcGasOctapoleMomentsYYZ	YYZ octapole moment from electrostatic potential charges in gas phase	Debye-Å^2
mepcGasOctapoleMomentsYZZ	YZZ octapole moment from electrostatic potential charges in gas phase	Debye-Å^2
mepcGasOctapoleMomentsZZZ	ZZZ octapole moment from electrostatic potential charges in gas phase	Debye-Å^2
mepcGasTracelessOctapoleXXX	XXX traceless octapole moment from electrostatic potential charges in gas phase	Debye-Å^2
mepcGasTracelessOctapoleYYY	YYY traceless octapole moment from electrostatic potential charges in gas phase	Debye-Å^2
mepcGasTracelessOctapoleZZZ	ZZZ traceless octapole moment from electrostatic potential charges in gas phase	Debye-Å^2
mepcGasTracelessOctapoleXYZ	XYZ traceless octapole moment from electrostatic potential charges in gas phase	Debye-Å^2
mepcGasTracelessOctapoleXXYYZZ	XXY-YZZ traceless octapole moment from electrostatic potential charges in gas phase	Debye-Å^2
mepcGasTracelessOctapoleXXZYYZ	XXZ-YYZ traceless octapole moment from electrostatic potential charges in gas phase	Debye-Å^2
mepcGasTracelessOctapoleXYYXZZ	XYY-XZZ traceless octapole moment from electrostatic potential charges in gas phase	Debye-Å^2
mepcGasHexadecapoleMomentsXXXX	XXXX hexadecapole moment from electrostatic potential charges in gas phase	Debye-Å^3
mepcGasHexadecapoleMomentsXXXY	XXXY hexadecapole moment from electrostatic potential charges in gas phase	Debye-Å^3
mepcGasHexadecapoleMomentsXXXZ	XXXZ hexadecapole moment from electrostatic potential charges in gas phase	Debye-Å^3
mepcGasHexadecapoleMomentsXXYY	XXYY hexadecapole moment from electrostatic potential charges in gas phase	Debye-Å^3
mepcGasHexadecapoleMomentsXXYZ	XXYZ hexadecapole moment from electrostatic potential charges in gas phase	Debye-Å^3
mepcGasHexadecapoleMomentsXXZZ	XXZZ hexadecapole moment from electrostatic potential charges in gas phase	Debye-Å^3
mepcGasHexadecapoleMomentsYYXZ	YYXZ hexadecapole moment from electrostatic potential charges in gas phase	Debye-Å^3
mepcGasHexadecapoleMomentsYYYX	YYYX hexadecapole moment from electrostatic potential charges in gas phase	Debye-Å^3
mepcGasHexadecapoleMomentsYYYY	YYYY hexadecapole moment from electrostatic potential charges in gas phase	Debye-Å^3
mepcGasHexadecapoleMomentsYYYZ	YYYZ hexadecapole moment from electrostatic potential charges in gas phase	Debye-Å^3
mepcGasHexadecapoleMomentsYYZZ	YYZZ hexadecapole moment from electrostatic potential charges in gas phase	Debye-Å^3
mepcGasHexadecapoleMomentsZZXY	ZZXY hexadecapole moment from electrostatic potential charges in gas phase	Debye-Å^3
mepcGasHexadecapoleMomentsZZZX	ZZZX hexadecapole moment from electrostatic potential charges in gas phase	Debye-Å^3
mepcGasHexadecapoleMomentsZZZY	ZZZY hexadecapole moment from electrostatic potential charges in gas phase	Debye-Å^3

mepcGasHexadecapoleMomentsZZZZ	ZZZZ hexadecapole moment from electrostatic potential charges in gas phase	Debye-Å^3
mepcDipoleMomentsTot	Total dipole moment from electrostatic potential charges in solution phase	Debye
mepcDipoleMomentsX	X dipole moment from electrostatic potential charges in solution phase	Debye
mepcDipoleMomentsY	Y dipole moment from electrostatic potential charges in solution phase	Debye
mepcDipoleMomentsZ	Z dipole moment from electrostatic potential charges in solution phase	Debye
mepcQuadrupoleMomentsXX	XX quadrupole moment from electrostatic potential charges in solution phase	Debye-Å
mepcQuadrupoleMomentsXY	XY quadrupole moment from electrostatic potential charges in solution phase	Debye-Å
mepcQuadrupoleMomentsXZ	XZ quadrupole moment from electrostatic potential charges in solution phase	Debye-Å
mepcQuadrupoleMomentsYY	YY quadrupole moment from electrostatic potential charges in solution phase	Debye-Å
mepcQuadrupoleMomentsYZ	YZ quadrupole moment from electrostatic potential charges in solution phase	Debye-Å
mepcQuadrupoleMomentsZZ	ZZ quadrupole moment from electrostatic potential charges in solution phase	Debye-Å
mepcTracelessQuadrupoleXY	XY traceless quadrupole moment from electrostatic potential charges in solution phase	Debye-Å
mepcTracelessQuadrupoleXZ	XZ traceless quadrupole moment from electrostatic potential charges in solution phase	Debye-Å
mepcTracelessQuadrupoleYZ	YZ traceless quadrupole moment from electrostatic potential charges in solution phase	Debye-Å
mepcTracelessQuadrupoleXXYY	XX-YY traceless quadrupole moment from electrostatic potential charges in solution phase	Debye-Å
mepcTracelessQuadrupol2ZZXXYY	2ZZ-XX-YY traceless quadrupole moment from electrostatic potential charges in solution phase	Debye-Å
mepcOctapoleMomentsXXX	XXX octapole moment from electrostatic potential charges in solution phase	Debye-Å^2
mepcOctapoleMomentsXXY	XXY octapole moment from electrostatic potential charges in solution phase	Debye-Å^2
mepcOctapoleMomentsXXZ	XXZ octapole moment from electrostatic potential charges in solution phase	Debye-Å^2
mepcOctapoleMomentsXYY	XYY octapole moment from electrostatic potential charges in solution phase	Debye-Å^2
mepcOctapoleMomentsXYZ	XYZ octapole moment from electrostatic potential charges in solution phase	Debye-Å^2
mepcOctapoleMomentsXZZ	XZZ octapole moment from electrostatic potential charges in solution phase	Debye-Å^2
mepcOctapoleMomentsYYY	YYY octapole moment from electrostatic potential charges in solution phase	Debye-Å^2
mepcOctapoleMomentsYYZ	YYZ octapole moment from electrostatic potential charges in solution phase	Debye-Å^2
mepcOctapoleMomentsYZZ	YZZ octapole moment from electrostatic potential charges in solution phase	Debye-Å^2
mepcOctapoleMomentsZZZ	ZZZ octapole moment from electrostatic potential charges in solution phase	Debye-Å^2
mepcTracelessOctapoleXXX	XXX traceless octapole moment from electrostatic potential charges in solution phase	Debye-Å^2

mepcTracelessOctapoleXYZ	XYZ traceless octapole moment from electrostatic potential charges in solution phase	Debye-Å^2
mepcTracelessOctapoleYYY	YYY traceless octapole moment from electrostatic potential charges in solution phase	Debye-Å^2
mepcTracelessOctapoleZZZ	ZZZ traceless octapole moment from electrostatic potential charges in solution phase	Debye-Å^2
mepcTracelessOctapoleXXYYZZ	XXY-YZZ traceless octapole moment from electrostatic potential charges in solution phase	Debye-Å^2
mepcTracelessOctapoleXXZYYZ	XXZ-YYZ traceless octapole moment from electrostatic potential charges in solution phase	Debye-Å^2
mepcTracelessOctapoleXYYXZZ	XYY-XZZ traceless octapole moment from electrostatic potential charges in solution phase	Debye-Å^2
mepcHexadecapoleMomentsXXXX	XXXX hexadecapole moment from electrostatic potential charges in solution phase	Debye-Å^3
mepcHexadecapoleMomentsXXXY	XXXY hexadecapole moment from electrostatic potential charges in solution phase	Debye-Å^3
mepcHexadecapoleMomentsXXXZ	XXXZ hexadecapole moment from electrostatic potential charges in solution phase	Debye-Å^3
mepcHexadecapoleMomentsXXYY	XXYY hexadecapole moment from electrostatic potential charges in solution phase	Debye-Å^3
mepcHexadecapoleMomentsXXYZ	XXYZ hexadecapole moment from electrostatic potential charges in solution phase	Debye-Å^3
mepcHexadecapoleMomentsXXZZ	XXZZ hexadecapole moment from electrostatic potential charges in solution phase	Debye-Å^3
mepcHexadecapoleMomentsYYXZ	YYXZ hexadecapole moment from electrostatic potential charges in solution phase	Debye-Å^3
mepcHexadecapoleMomentsYYYX	YYYX hexadecapole moment from electrostatic potential charges in solution phase	Debye-Å^3
mepcHexadecapoleMomentsYYYY	YYYY hexadecapole moment from electrostatic potential charges in solution phase	Debye-Å^3
mepcHexadecapoleMomentsYYYZ	YYYZ hexadecapole moment from electrostatic potential charges in solution phase	Debye-Å^3
mepcHexadecapoleMomentsYYZZ	YYZZ hexadecapole moment from electrostatic potential charges in solution phase	Debye-Å^3
mepcHexadecapoleMomentsZZXY	ZZXY hexadecapole moment from electrostatic potential charges in solution phase	Debye-Å^3
mepcHexadecapoleMomentsZZZX	ZZZX hexadecapole moment from electrostatic potential charges in solution phase	Debye-Å^3
mepcHexadecapoleMomentsZZZY	ZZZY hexadecapole moment from electrostatic potential charges in solution phase	Debye-Å^3
mepcHexadecapoleMomentsZZZZ	ZZZZ hexadecapole moment from electrostatic potential charges in solution phase	Debye-Å^3
mmcGasDipoleMomentsTot	Total dipole moments from Mulliken charges in gas phase	Debye
mmcGasDipoleMomentsX	X dipole moments from Mulliken charges in gas phase	Debye
mmcGasDipoleMomentsY	Y dipole moments from Mulliken charges in gas phase	Debye
mmcGasDipoleMomentsZ	Z dipole moments from Mulliken charges in gas phase	Debye
mmcGasQuadrupoleMomentsXX	XX quadrupole moments from Mulliken charges in gas phase	Debye-Å
mmcGasQuadrupoleMomentsXY	XY quadrupole moments from Mulliken charges in gas phase	Debye-Å

mmcGasQuadrupoleMomentsXZ	XZ quadrupole moments from Mulliken charges in gas phase	Debye-Å
mmcGasQuadrupoleMomentsYY	YY quadrupole moments from Mulliken charges in gas phase	Debye-Å
mmcGasQuadrupoleMomentsYZ	YZ quadrupole moments from Mulliken charges in gas phase	Debye-Å
mmcGasQuadrupoleMomentsZZ	ZZ quadrupole moments from Mulliken charges in gas phase	Debye-Å
mmcGasTracelessQuadrupoleXY	XY traceless quadrupole moments from Mulliken charges in gas phase	Debye-$Å^2$
mmcGasTracelessQuadrupoleXZ	XZ traceless quadrupole moments from Mulliken charges in gas phase	Debye-$Å^2$
mmcGasTracelessQuadrupoleYZ	YZ traceless quadrupole moments from Mulliken charges in gas phase	Debye-$Å^2$
mmcGasTracelessQuadrupoleXXYY	XX-YY traceless quadrupole moments from Mulliken charges in gas phase	Debye-$Å^2$
mmcGasTracelessQuadrupol2ZZXXYY	2ZZ-XX-YY traceless quadrupole moments from Mulliken charges in gas phase	Debye-$Å^2$
mmcGasOctapoleMomentsXXX	XXX octapole moments from Mulliken charges in gas phase	Debye-$Å^2$
mmcGasOctapoleMomentsXXY	XXY octapole moments from Mulliken charges in gas phase	Debye-$Å^2$
mmcGasOctapoleMomentsXXZ	XXZ octapole moments from Mulliken charges in gas phase	Debye-$Å^2$
mmcGasOctapoleMomentsXYY	XYY octapole moments from Mulliken charges in gas phase	Debye-$Å^2$
mmcGasOctapoleMomentsXYZ	XYZ octapole moments from Mulliken charges in gas phase	Debye-$Å^2$
mmcGasOctapoleMomentsXZZ	XZZ octapole moments from Mulliken charges in gas phase	Debye-$Å^2$
mmcGasOctapoleMomentsYYY	YYY octapole moments from Mulliken charges in gas phase	Debye-$Å^2$
mmcGasOctapoleMomentsYYZ	YYZ octapole moments from Mulliken charges in gas phase	Debye-$Å^2$
mmcGasOctapoleMomentsYZZ	YZZ octapole moments from Mulliken charges in gas phase	Debye-$Å^2$
mmcGasOctapoleMomentsZZZ	ZZZ octapole moments from Mulliken charges in gas phase	Debye-$Å^2$
mmcGasTracelessOctapoleXXX	XXX traceless octapole moments from Mulliken charges in gas phase	Debye-$Å^2$
mmcGasTracelessOctapoleXYZ	XYZ traceless octapole moments from Mulliken charges in gas phase	Debye-$Å^2$
mmcGasTracelessOctapoleYYY	YYY traceless octapole moments from Mulliken charges in gas phase	Debye-$Å^2$
mmcGasTracelessOctapoleZZZ	ZZZ traceless octapole moments from Mulliken charges in gas phase	Debye-$Å^2$
mmcGasTracelessOctapoleXXYYZZ	XXY-YZZ traceless octapole moments from Mulliken charges in gas phase	Debye-$Å^2$
mmcGasTracelessOctapoleXXZYYZ	XXZ-YYZ traceless octapole moments from Mulliken charges in gas phase	Debye-$Å^2$
mmcGasTracelessOctapoleXYYXZZ	XYY-XZZ traceless octapole moments from Mulliken charges in gas phase	Debye-$Å^2$
mmcGasHexadecapoleMomentsXXXX	XXXX hexadecapole moments from Mulliken charges in gas phase	Debye-$Å^3$
mmcGasHexadecapoleMomentsXXXY	XXXY hexadecapole moments from Mulliken charges in gas phase	Debye-$Å^3$

mmcGasHexadecapoleMomentsXXXZ	XXXZ hexadecapole moments from Mulliken charges in gas phase	Debye-Å^3
mmcGasHexadecapoleMomentsXXYY	XXYY hexadecapole moments from Mulliken charges in gas phase	Debye-Å^3
mmcGasHexadecapoleMomentsXXYZ	XXYZ hexadecapole moments from Mulliken charges in gas phase	Debye-Å^3
mmcGasHexadecapoleMomentsXXZZ	XXZZ hexadecapole moments from Mulliken charges in gas phase	Debye-Å^3
mmcGasHexadecapoleMomentsYYXZ	YYXZ hexadecapole moments from Mulliken charges in gas phase	Debye-Å^3
mmcGasHexadecapoleMomentsYYYX	YYYX hexadecapole moments from Mulliken charges in gas phase	Debye-Å^3
mmcGasHexadecapoleMomentsYYYY	YYYY hexadecapole moments from Mulliken charges in gas phase	Debye-Å^3
mmcGasHexadecapoleMomentsYYYZ	YYYZ hexadecapole moments from Mulliken charges in gas phase	Debye-Å^3
mmcGasHexadecapoleMomentsYYZZ	YYZZ hexadecapole moments from Mulliken charges in gas phase	Debye-Å^3
mmcGasHexadecapoleMomentsZZXY	ZZXY hexadecapole moments from Mulliken charges in gas phase	Debye-Å^3
mmcGasHexadecapoleMomentsZZZX	ZZZX hexadecapole moments from Mulliken charges in gas phase	Debye-Å^3
mmcGasHexadecapoleMomentsZZZY	ZZZY hexadecapole moments from Mulliken charges in gas phase	Debye-Å^3
mmcGasHexadecapoleMomentsZZZZ	ZZZZ hexadecapole moments from Mulliken charges in gas phase	Debye-Å^3
mmcDipoleMomentsTot	Total dipole moments from Mulliken charges in solution phase	Debye
mmcDipoleMomentsX	X dipole moments from Mulliken charges in solution phase	Debye
mmcDipoleMomentsY	Y dipole moments from Mulliken charges in solution phase	Debye
mmcDipoleMomentsZ	Z dipole moments from Mulliken charges in solution phase	Debye
mmcQuadrupoleMomentsXX	XX quadrupole moments from Mulliken charges in solution phase	Debye-Å
mmcQuadrupoleMomentsXY	XY quadrupole moments from Mulliken charges in solution phase	Debye-Å
mmcQuadrupoleMomentsXZ	XZ quadrupole moments from Mulliken charges in solution phase	Debye-Å
mmcQuadrupoleMomentsYY	YY quadrupole moments from Mulliken charges in solution phase	Debye-Å
mmcQuadrupoleMomentsYZ	YZ quadrupole moments from Mulliken charges in solution phase	Debye-Å
mmcQuadrupoleMomentsZZ	ZZ quadrupole moments from Mulliken charges in solution phase	Debye-Å
mmcTracelessQuadrupoleXY	XY traceless quadrupole moments from Mulliken charges in solution phase	Debye-Å
mmcTracelessQuadrupoleXZ	XZ traceless quadrupole moments from Mulliken charges in solution phase	Debye-Å
mmcTracelessQuadrupoleYZ	YZ traceless quadrupole moments from Mulliken charges in solution phase	Debye-Å
mmcTracelessQuadrupoleXXYY	XX-YY traceless quadrupole moments from Mulliken charges in solution phase	Debye-Å
mmcTracelessQuadrupol2ZZXXYY	2ZZ-XX-YY traceless quadrupole moments from Mulliken charges in solution phase	Debye-Å

mmcOctapoleMomentsXXX	XXX octapole moments from Mulliken charges in solution phase	Debye-Å^2
mmcOctapoleMomentsXXY	XXY octapole moments from Mulliken charges in solution phase	Debye-Å^2
mmcOctapoleMomentsXXZ	XXZ octapole moments from Mulliken charges in solution phase	Debye-Å^2
mmcOctapoleMomentsXYY	XYY octapole moments from Mulliken charges in solution phase	Debye-Å^2
mmcOctapoleMomentsXYZ	XYZ octapole moments from Mulliken charges in solution phase	Debye-Å^2
mmcOctapoleMomentsXZZ	XZZ octapole moments from Mulliken charges in solution phase	Debye-Å^2
mmcOctapoleMomentsYYY	YYY octapole moments from Mulliken charges in solution phase	Debye-Å^2
mmcOctapoleMomentsYYZ	YYZ octapole moments from Mulliken charges in solution phase	Debye-Å^2
mmcOctapoleMomentsYZZ	YZZ octapole moments from Mulliken charges in solution phase	Debye-Å^2
mmcOctapoleMomentsZZZ	ZZZ octapole moments from Mulliken charges in solution phase	Debye-Å^2
mmcTracelessOctapoleXXX	XXX traceless octapole moments from Mulliken charges in solution phase	Debye-Å^2
mmcTracelessOctapoleXYZ	XYZ traceless octapole moments from Mulliken charges in solution phase	Debye-Å^2
mmcTracelessOctapoleYYY	YYY traceless octapole moments from Mulliken charges in solution phase	Debye-Å^2
mmcTracelessOctapoleZZZ	ZZZ traceless octapole moments from Mulliken charges in solution phase	Debye-Å^2
mmcTracelessOctapoleXXYYZZ	XXY-YZZ traceless octapole moments from Mulliken charges in solution phase	Debye-Å^2
mmcTracelessOctapoleXXZYYZ	XXZ-YYZ traceless octapole moments from Mulliken charges in solution phase	Debye-Å^2
mmcTracelessOctapoleXYYXZZ	XYY-XZZ traceless octapole moments from Mulliken charges in solution phase	Debye-Å^2
mmcHexadecapoleMomentsXXXX	XXXX hexadecapole moments from Mulliken charges in solution phase	Debye-Å^3
mmcHexadecapoleMomentsXXXY	XXXY hexadecapole moments from Mulliken charges in solution phase	Debye-Å^3
mmcHexadecapoleMomentsXXXZ	XXXZ hexadecapole moments from Mulliken charges in solution phase	Debye-Å^3
mmcHexadecapoleMomentsXXYY	XXYY hexadecapole moments from Mulliken charges in solution phase	Debye-Å^3
mmcHexadecapoleMomentsXXYZ	XXYZ hexadecapole moments from Mulliken charges in solution phase	Debye-Å^3
mmcHexadecapoleMomentsXXZZ	XXZZ hexadecapole moments from Mulliken charges in solution phase	Debye-Å^3
mmcHexadecapoleMomentsYYXZ	YYXZ hexadecapole moments from Mulliken charges in solution phase	Debye-Å^3
mmcHexadecapoleMomentsYYYX	YYYX hexadecapole moments from Mulliken charges in solution phase	Debye-Å^3
mmcHexadecapoleMomentsYYYY	YYYY hexadecapole moments from Mulliken charges in solution phase	Debye-Å^3
mmcHexadecapoleMomentsYYYZ	YYYZ hexadecapole moments from Mulliken charges in solution phase	Debye-Å^3
mmcHexadecapoleMomentsYYZZ	YYZZ hexadecapole moments from Mulliken charges in solution phase	Debye-Å^3

	mmcHexadecapoleMomentsZZXY	ZZXY hexadecapole moments from Mulliken charges in solution phase	Debye-Å^3
	mmcHexadecapoleMomentsZZZX	ZZZX hexadecapole moments from Mulliken charges in solution phase	Debye-Å^3
	mmcHexadecapoleMomentsZZZY	ZZZY hexadecapole moments from Mulliken charges in solution phase	Debye-Å^3
	mmcHexadecapoleMomentsZZZZ	ZZZZ hexadecapole moments from Mulliken charges in solution phase	Debye-Å^3

Table S2: Explanation of the data columns that are present in RedDB's additional data tables. Descriptions of data columns have been provided alongside the units, whenever applicable, for the different data types.
